# Cryptic Species Diversity and Phylogenetic Relationship in the Rust Genus *Chrysomyxa* from China

**DOI:** 10.3390/jof8010083

**Published:** 2022-01-15

**Authors:** Rui Wang, Clement K. M. Tsui, Chongjuan You

**Affiliations:** 1 Beijing Key Laboratory for Forest Pest Control, College of Forestry, Beijing Forestry University, Beijing 100083, China; wangrui_0121@bjfu.edu.cn; 2 Department of Pathology, Sidra Medicine, Doha 2713, Qatar; clementsui@gmail.com; 3 Department of Pathology and Laboratory Medicine, Weill Cornell Medicine-Qatar, Doha 2713, Qatar; 4 Division of Infectious Diseases, Faculty of Medicine, University of British Columbia, Vancouver, BC V6T 1Z3, Canada

**Keywords:** rust fungus, cryptic species, *Chrysomyxa*, biodiversity, taxonomy, species delimitation

## Abstract

*Chrysomyxa* rusts are fungal pathogens widely distributed in the Northern hemisphere, causing spruce needle and cone rust diseases, and they are responsible for significant economic losses in China. Taxonomic delimitation and precise species identification are difficult within this genus because some characters often overlap in several species. Adequate species delimitation, enhanced by the use of DNA-based methodologies, will help to establish well-supported species boundaries and enable the identification of cryptic species. Here, we explore the cryptic species diversity in the rust genus *Chrysomyxa* from China. Species delimitation analyses are conducted using a distance-based method (ABGD) and three tree-based methods (GMYC, bPTP, and mPTP) based on combined LSU and ITS sequences of over 60 specimens. Although there is some incongruence among species delimitation methods, two new species and three putative cryptic species are identified. The key to 20 *Chrysomyxa* species distributed in China is presented. These results suggest that a significant level of undiscovered cryptic diversity is likely to be found in *Chrysomyxa* from China. Future studies should consider multiple analytical methods when dealing with multi-locus datasets.

## 1. Introduction

Rust fungi (Pucciniomycotina, Pucciniales) are some of the most important plant pathogens that cause devastating diseases of agricultural crops and forests. There are approximately 8000 described species, which makes it the largest and most diverse group in Basidiomycota, the members of which are usually obligate parasites and host-specific [1,2,3]. Rust fungi have complex and variable life cycles that include up to five different spore stages (spermatia, aeciospores, urediniospores, teliospores, and basidiospores) on a single host or two unrelated hosts [4]. Rust taxonomy has traditionally been based on a combination of host specificities and morphological traits of certain spore stages, which are potentially plastic [5,6,7]. Several studies have uncovered a great number of rust species complexes and cryptic species. For example, 14 different phylogenetic species were determined in the *Melampsora epitea* complex [8]. Similar patterns of cryptic speciation have also been in the rust species *Endoraecium digitatum* and *Dasyspora gregaria* [9,10,11].

Rust fungi of the genus *Chrysomyxa* Unger (Coleosporiaceae, Pucciniales) are widespread in the Northern hemisphere, causing spruce needle and cone rust diseases, which are responsible for significant economic losses, especially in spruce plantations and natural forests in northwest and southwest China [12,13,14,15,16]. Most *Chrysomyxa* species are heteroecious, macrocyclic and they produce the spermogonia and aecia on Pinaceae, while also the uredinia and telia on Ericaceae and Pyrolaceae, mostly on *Rhododendron* [2,4,12,15,16]. The monophyly of *Chrysomyxa* was supported by LSU sequence data from two closely related *Chrysomyxa* species (*Chrysomyxa ledi* and *C. rhododendri*) [17]. However, *Chrysomyxa* was indicated to be polyphyletic based on a higher-rank molecular systematic framework of two or three DNA loci. Both Maier et al. [17] and Feau et al. [15] demonstrated that the autoecious microcyclic species *C. weirii* clustered with the genus *Melampsora* and the spruce cone rusts *C. pyrolae* and *C. monesis* coalesced with the pine needle rusts belonging to the genus *Coleosporium*. Aime et al. [2] proposed a new genus *Rossmanomyces* for *C. monesis* and *C. pyrolae*, and they also determined that *C. weirii* should be placed in *Ceropsora*, which was characterized by forming laterally adherent teliospores, germinating to produce two-celled basidia.

The species delimitation of *Chrysomyxa* has been controversial as several species display similar morphological features; the traditional identification is mainly based on host specificities and morphological characteristics of each spore stage [12,15,16,18]. For example, *C. rhododendri* on *Rhododendron* in Europe and *C. ledi* on *Ledum* in North America have subtle morphological and ecological traits, but they have distinct host specificities [15,19]. Some *Chrysomyxa* species exhibit intraspecific morphological variation across their geographic ranges suggesting the existence of cryptic species. Feau et al. [15] observed genetically distinct lineages within *C. pyrolae* and *C. rhododendri*, providing some evidence for allopatric speciation within the two morphologically defined species. Moreover, our earlier phylogenetic analyses based on two genetic markers indicated that *C. rhododendri* from North America, China, and Europe possibly contained cryptic species, which form distinct phylogenetic lineages but cannot be easily distinguished based on morphologies [13,14,16].

Adequate species delimitation, enhanced by the use of DNA-based methodologies, will help to establish well-supported species boundaries and enable the identification of cryptic species [20,21,22]. A variety of methods for delimiting fungal species have been developed and applied, for example, the Generalized Mixed Yule Coalescent (GMYC) [23], Automatic Barcode Gap Discovery (ABGD) [24], Multi-rate Poisson Tree Processes (mPTP) [25], and Bayesian implementation of the PTP model (bPTP) [26]. These approaches offer the opportunity to delineate species in a comparative way. However, Maharachchikumbura et al. [22] indicated that discordance between the boundaries of putative species inferred by different delimitation analyses can lead to uncertainty in genetic diversity estimates due to either over-splitting or over-lumping the taxa. The integration of multi-locus molecular analyses with morphological examination and coalescence-based methods is needed [27,28]

The objectives of our investigation are to analyze the species boundaries of *Chrysomyxa* in China, and to infer the placement of potential cryptic species by using a polyphasic methodology that compares morphological characteristics and integrative analyses of genetic distance (ABGD) and coalescent (GMYC, bPTP and mPTP) approaches for two markers (LSU and ITS). Our work will characterize the cryptic species diversity of the genus *Chrysomyxa* and clarify the methods and characters that are most useful for distinguishing otherwise “cryptic” species in *Chrysomyxa*. The data will also shed light on the utility of molecular species delimitation in the evolutionary study of closely related rust species, and may contribute to a better understanding of rust species diversity in China.

## 2. Materials and Methods

### 2.1. Sample Collections

A total of 452 fresh rust specimens were collected from China between 2014–2020 and they were deposited at Mycological Herbarium, Museum of Beijing Forestry University, Beijing, China (BJFC). Dried specimens borrowed from Herbarium Mycologicum Academiae Sinicae, Beijing (HMAS), were also included in this study. Host plants, locality of collection, and accession numbers for sequence data from GenBank and Barcode of Life Database (BOLD, www.barcodinglife.org, accessed on 1 September 2021) are listed in Supplementary Table S1.

### 2.2. Morphological Analyses

Morphological characters of studied specimens were compared to the type specimens and original descriptions [4,12,13,14,15,16,29,30,31]. For each specimen, approximately 50–60 spores were mounted in a drop of lactophenol or lactophenol cotton blue solution on a microscopic slide and randomly measured. The spore size and shape were determined using an Olympus SZX2-FOF Light microscope (Tokyo, Japan). To prepare samples for surface structure examination using scanning electron microscopy (SEM), telium, aeciospores, and urediniospores were adhered onto aluminum stubs covered with a double-sided adhesive tape and gold-coated using the SCD-005 Sputter Coater. Then, observation was done using a Hitachi SU8010 scanning electron microscope (Tokyo, Japan) operated at 3.0 Kv [16].

### 2.3. DNA Extraction

Genomic DNA was extracted from fresh specimens using a QIAamp DNA Microbiome kit (Qiagen, Hilden, Germany) in accordance with the manufacturer’s instructions. Two nuclear ribosomal RNA gene regions: the internal transcribed spacer regions and intervening 5.8S nrRNA gene (ITS), and the large subunit (LSU) rDNA, were amplified. The primer sequences and the methods and conditions for PCR amplification and sequencing were described in You et al. [16].

### 2.4. Phylogenetic Analyses

Consensus ITS and LSU sequences were edited using Seqman v. 7.1.0 in the DNASTAR Lasergene core suite software (Madison, WI, USA) [32] and aligned using MAFFT v. 6.0.0 [33]. Ambiguously aligned sequences were excluded from the analysis. Data analysis for molecular biology and evolution (DAMBE) was used to test for possible substitution saturation and plotted transitions and transversions against K2P distances [34,35]. Maximum parsimony (MP) analysis of concatenated ITS and LSU alignment was performed in PAUP v.4.0b10 [36]. Gaps were treated as missing data, and all characters were equally weighted. Trees were inferred using the heuristic search option with tree bisection and reconnection (TBR) branch swapping. The robustness of the most parsimonious trees was evaluated by 1000 bootstrap replications [37]. Other calculated measures were the tree length (TL), consistency index (CI), retention index (RI), and rescaled consistency (RC).

Bayesian analyses (BI) were performed using MrBayes v. 3.1.2 with Markov Chain Monte Carlo (MCMC), and Bayesian posterior probabilities were estimated [38]. A jModelTest v. 2.1.7 [39,40] was used to determine the best-fit model for each dataset; the models selected were TPM2uf+G model for ITS, and TIM1+G model for LSU. Simultaneous Markov chains were run for 10,000,000 generations, and trees were sampled every 1000th generation. The first 2000 trees, representing the burn-in phase of the analyses, were discarded and the remaining 8000 trees were used to calculate PPs in the majority rule consensus tree [16].

Maximum likelihood (ML) analyses were run with IQ-TREE v.1.6.5 [41], using models selected by ModelFinder [42] for each partitioned gene; ultrafast bootstrap (BS) analysis with 1000 replicates estimated branch support in the ML tree [43]. All trees were visualized with FigTree (http://tree.bio.ed.ac.uk/software/figtree/). *Melampsora epitea* was selected as the outgroup.

### 2.5. Species Delimitation Analyses

We tested one distance-based method (ABGD) and three tree-based methods (GMYC, bPTP, and mPTP) of species delimitation. The dataset employed for all methods consisted of concatenated ITS and LSU sequences from 28 putative *Chrysomyxa* species and one outgroup (*M. epitea*). ABGD analyses were performed on the online server (https://bioinfo.mnhn.fr/abi/public/abgd/abgdweb.html) [24], using the Kimura two-parameter substitution model, prior for maximum value of intraspecific divergence between 0.001 and 0.1, and a minimum gap width (X) of 0.8. After the calculation was completed, the corresponding genetic distance frequency distribution histogram, genetic distance grading curve, and preliminary initial division and recursive division results were obtained [28].

bPTP analyses, which assume independent exponential distributions to model the branch lengths for speciation and for coalescence, were conducted on the online server (http://species.h-its.org) using the maximum-likelihood tree from IQ-TREE [26]. Each analysis consisted of 500,000 generations, with a thinning every 500 generations and a burn-in proportion of 0.1. The recently introduced multi-rate PTP (mPTP), delimits species assuming a constant speciation rate with different intraspecific coalescent rates [25]. For this analysis, an ML tree was used as input on the web server (http://mptp.h-its.org/#/tree). We ran two independent Markov Chain Monte Carlo (MCMC) analyses for 100 million steps, sampling every 10,000 steps, to assess Average Support Values (ASV) and the confidence of the ML delimitation for each species [28].

GMYC model distinguishes between intraspecific (coalescent process) and interspecific (Yule process) branching events on a phylogenetic tree [44]. The summarized ultrametric trees, created in BEAST ver.2.4.8.0., were required to rim the GMYC algorithm [45]. Two independent MCMC analyses were run for 100 million generations, sampling trees every 100,000 generations. The posterior tree was summarized using TreeAnnotator ver.2.4.8.0 after discarding burn-in, which has been determined by Tracer ver.1.7 [44,46]. The single threshold and multiple threshold models were optimized, using the “splits” package for R v3.6.3 [47]. Finally, the putative species scenario was selected based on the value of estimated likelihood scores.

In accordance with Ahrens et al. [48], Blair et al. [27], and Hofmann et al. [28], the match ratio was used to provide a better comparable value for different delimitation analyses, where splitting and lumping species cancels out match value.

The match ratio was calculated as follows:

$$\mathrm{Match}\;\mathrm{ratio}=2\times \frac{N_{match}}{N_{delimited}-N_{morph}}$$

*N_match_* refers to the number of delimited species that exactly match a taxonomically defined morphospecies (not including taxa split as cryptic lineages), *N_delimited_* refers to the total number of lineages delimited by an analysis, and *N_morph_* refers to the total number of morphologically defined species.

## 3. Results

### 3.1. Phylogenetic Relationships

The concatenated ITS and LSU sequence datasets consisted of 68 *Chrysomyxa* ingroup of 699 base pairs, which when aligned were 1177 characters in length including gaps, and with 734 monomorphic characters, and 205 characters were variable and parsimony informative.

Substitution saturation of the concatenated LSU and ITS sequences on all codon positions were tested by DAMBE. The results showed that the index of substitution saturation (Iss) was less than the critical index of substitution saturation assuming a symmetrical topology (Issc. Sym) or critical index of substitution saturation assuming an asymmetrical topology (Issc. Asym) for all numbers of species simulated (NumOTU), except for a higher Iss value than Issc. Asym for NumOTU 32 (Table 1), suggesting little substitution saturation had occurred [35].

**Figure 1:**
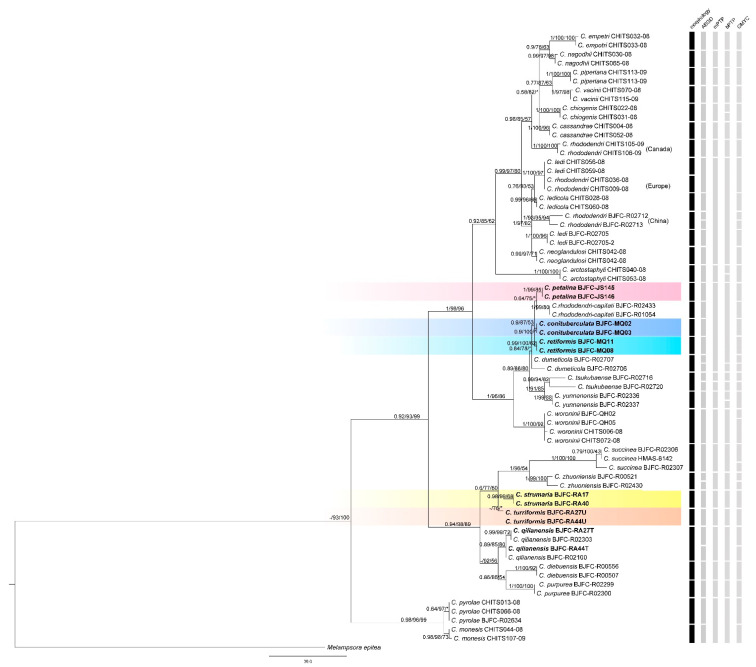
Phylogenetic analysis of *Chrysomyxa* species inferred from the concatenated alignment by maximum parsimony, maximum likelihood, and Bayesian analyses based on ITS+LSU rDNA dataset. The topology is the result of maximum parsimony inference performed with win-paup4b10. Bootstrap values were calculated from 1000 replications. Bootstrap value ≥ 50% for BI, ML, and MP analyses are presented at the first, second, and third position, which is BI/ML/MP. The ‘*’ means bootstrap value < 50%, and ‘-’ indicates that the support rate of the node is not given in the BI or ML topology. Scale bar = 30.0 nucleotide substitutions. The new species of *Chrysomyxa* are highlighted in bold, and the different colored rectangular gradients represent the different new species of *Chrysomyxa* parasitic on *Rhododendron* or *Picea.* The first column depicts species recognized by morphological taxonomy. The second to fifth columns depict putative species recognized by the recursive partition (*p* = 0.001) of ABGD, mPTP, bPTP and single-threshold GMYC model, respectively.

Tree topologies based on BI, ML, and MP inference were congruent. All studied *Chrysomyxa* species were recovered as a single group, with the only exception being *C. pyrolae* and *C. monesis,* which were recovered as sister taxa to all the other *Chrysomyxa* species with high support (0.98/91/99, Figure 1). Aime et al. [2] also assigned the two species to new genera *Rossmanomyces.* The rest of the in-group *Chrysomyxa* species clustered into two independent clades. The first monophyletic clade consisted of two subclades of samples of China, North American, and European origin, respectively. The second clade was composed of five accepted *Chrysomyxa* species (*C. succinea*, *C. zhuoniensis*, *C. qilianensis*, *C. diebuensis*, and *C. purpurea*), and two putative cryptic species, all of which were only recorded in Asia.

### 3.2. Phylogenetic Relationships

Tree- and distance-based methods of species delimitation did not produce congruent results and resolved different numbers of species (33, 21, 25, and 35 in ABGC, mPTP, bPTP, and GMYC, respectively, Figure 1). The differences between species numbers estimated by three tree-based methods were very variable: GMYC recovered a higher number of species and overestimated the number of lineages, while mPTP underestimated it. ABGD match ratios were the highest of all analyses (0.90, Table 2), while GMYC match ratios were highest among tree-based analyses (0.84, Table 2). Despite these differences, species number estimations estimated by ABGD were closer to the number of species morphologically identified, and the number of lineages found by bPTP and mPTP was lower than those identified by morphology. Nevertheless, 12 putative species were recognized by all four of the methods, and these are supported by morphological diagnosis in previous taxonomic studies [13,14,15,16].

According to all species delimitation analyses, *C. rhododendri* from North America, Europe, and China were separated as three hypothetical species in GMYC and ABGD, and *C. ledi* from North America (CHITS056-08, CHITS059-08) and China (BJFC-R02705, BJFC-R02705-2) were divided into two candidate species in ABGD and GMYC. In addition, GMYC and ABGD analyses delimited two operational taxonomic units (OTUs) within some morphologically identified species, for example, *C. qilianensis*, *C. zhuoniensis*, *C. dumeticola*, and *C. rhododendri* from China. However, no evident host, spore-stage or geographic association was found to be related to the differentiation of these two OTUs. ABGD and GMYC analyses also showed that *C. ledi* from Canada (CHITS056-08, CHITS059-08) and *C. rhododendri* from Europe (CHITS036-08, CHITS009-08), as a well-defined sister clade to *C. ledicola*, should be considered as conspecific.

mPTP and bPTP analyses grouped these following species: *C. ledi*, *C. ledicola*, *C. neoglandulosi* from Canada, *C. rhododendri* from Europe, C. *rhododendri* and *C. ledi* from China, into a single species. Moreover, two new species, *C. strumarium* and *C. turrigormis*, were delimited by all of four of the analyses, differed from other known *Chrysomyxa* species in having unique urediniospore morphology. Three candidate cryptic species, *C. petalinus*, *C. conimmamus*, and *C. retiformis*, were recognized by ABGD and GMYC analyses, but mPTP and bPTP analyses failed to delimit them from *C. rhododendri-capitati*.

### 3.3. Taxonomic Implications

Based on the comparison of the multiple methods for species delimitation that we applied to the concatenated ITS and LSU dataset, we consider ABGD to be the most reasonable distance-based estimate and GMYC to be the most reasonable tree-based methods of delimitation for this dataset. These methods inferred 33 and 35 lineages, respectively, and yielded largely congruent estimates of species boundaries based on morphology. Two new species, *C. strumaria* and *C. turriformis*, were supported by every analysis, and three candidate cryptic species were recognized by ABGD and GMYC analyses. Expanded descriptions and illustrations of the five species were provided, and the key to 20 *Chrysomyxa* species distributed in China was given.

**Figure 2:**
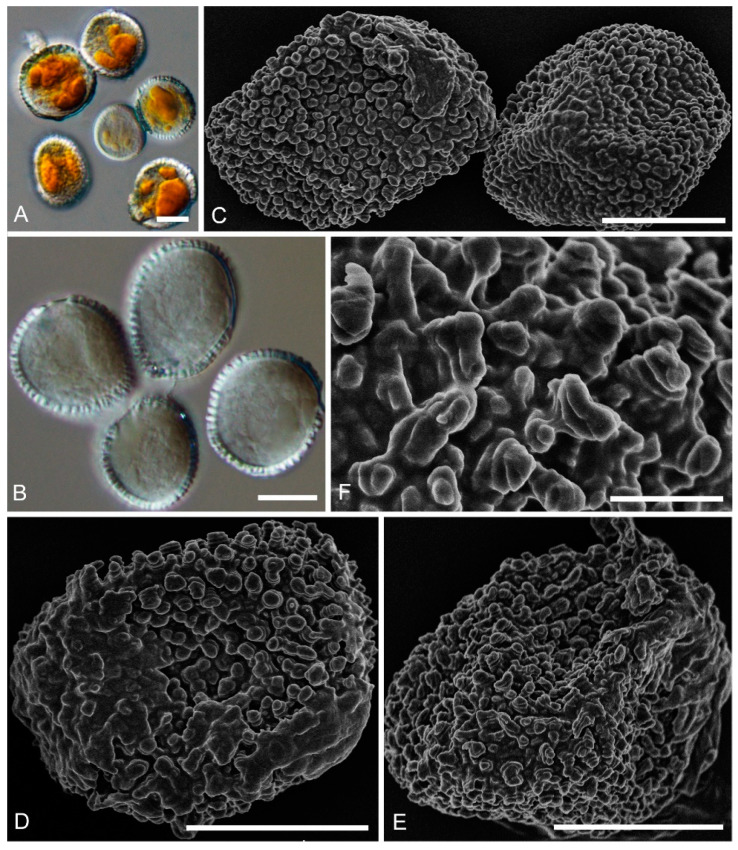
Light and electron micrographs of *Chrysomyxa strumaria*. (BJFC-RA17). (**A**,**B**) Globose to subglobose urediniospores observed by DIC. (**C**) Urediniospores. (**D**,**E**) Single urediniospore, densely covered with warts. (**F**) Details of ornamentation. Scale bars: (**A**–**E**) = 10 μm; (**F**) = 2 μm.

***Chrysomyxastrumaria*** C. J. You and R. Wang, **sp. nov.** (Figure 2)

**MycoBank:** MB 840239

**Specimens examined:** CHINA, Qinghai Province: Huzhu County, On *Rhododendron przewalskii* Maxim., 36°51′42″ N and 102°34′20″ E, 3120 m asl., 30 August 2020, coll. (BJFC-RA17; BJFC-RA40).

**Etymology:** Name refers to the struma-like warts on the urediniospore surface.

Spermogonia, aecia and telia: Unknown.

**Descriptions:** *Uredinia* on *R. przewalskii*, Uredinia hypophyllous, scattered or in groups. Urediniospores oval, globose to subglobose, 17–25 × 15–24 μm, with wall 0.7–1.5 μm thick (Figure 2A,B); densely warted, warts annulate, struma-like, some warts fused (Figure 2D–F).

**Notes:***Chrysomyxa strumaria* is represented by two specimens on *R. przewalskii*, which cluster in a distinct clade in *Chrysomyxa* genus and is clearly distinguished from other known *Chrysomyxa* species (Figure 1). *C.* *strumaria* is distinct from *C. zhuoniensis* by its fused struma-like warts on the urediniospores surface and smaller urediniospore.

**Figure 3:**
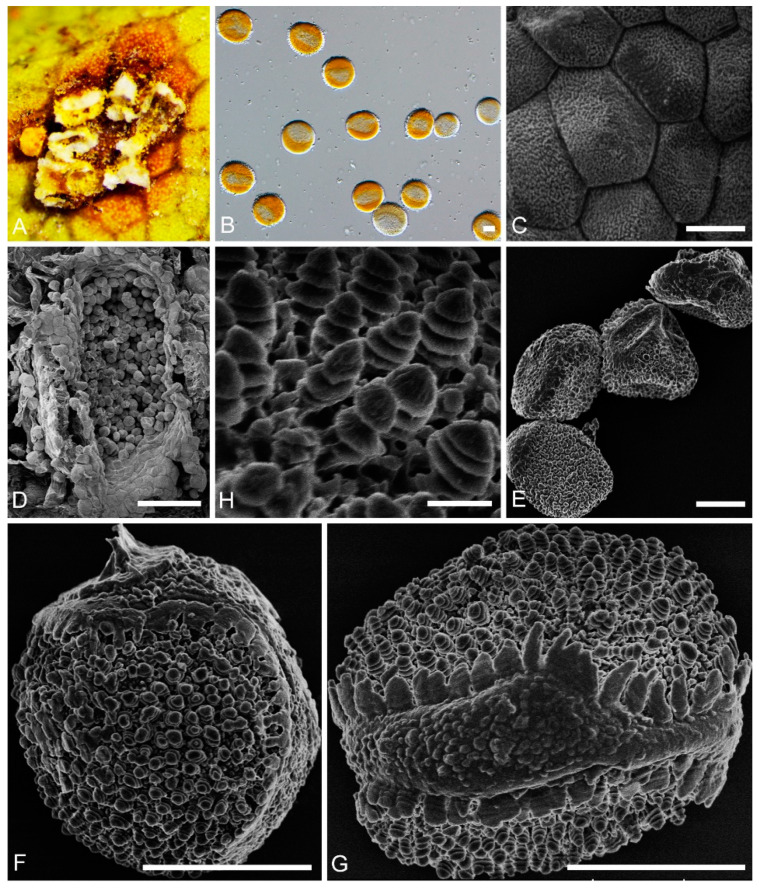
Light and electron micrographs of *Chrysomyxa turriformis*. (BJFC-RA44U). (**A**) Uredinia observed by SEM. (**B**) Urediniospores observed by DIC. (**C**) Uredinia peridium with densely warted inner surface. (**D**) Uredinia circular or elliptical. (**E**) Urediniospores. (**F**, **G**) Single urediniospore, showing broad longitudinal area or cap, serrated edge. (**H**) Urediniospore with conical annulate. Scale bars: (**B**,**C**) = 10 μm; (**E**,**F**) = 10 μm; (**D**) = 100 μm; (**H**) = 1 μm.

***Chrysomyxa turriformis*** C. J. You and R. Wang, **sp. nov.** (Figure 3)

**MycoBank:** MB 840240

**Specimens examined:** CHINA, Qinghai Province: Huzhu County, On *Rhododendron przewalskii* Maxim., 36°51′44″ N and 102°34′21″ E, 3226 m asl., 30 August 2020, coll. (BJFC-RA27U; BJFC-RA44U).

**Etymology:** Name refers to the tower-like warts on the urediniospore surface.

Spermogonia, aecial and telia: unknown.

**Descriptions:***Uredinia* on *R. przewalskii*. Uredinia hypophyllous, scattered, or crowded in groups, covered by a conspicuous peridium (Figure 3A); Uredinia peridium polygonal, inner surface shallowly concave, densely warted (Figure 3C); Urediniospores globose to subglobose, 30–40 × 25–35 μm (Figure 3B), with a narrow shallow cap at one or both ends, part of a broad rounded area with a broken, skirt-like edge (Figure 3F,G); warts crowded, annulate, tower-like, with smooth and rounded tops (Figure 3H).

**Notes:***Chrysomyxa turriformis* formed an independent clade in the phylogenetic tree of *Chrysomyxa* (Figure 1). Morphologically, it is characterized by globose urediniospore with a narrower and bumped cap, and tower-like warts with rounded tops on spore surface, which distinguished it from other *Chrysomyxa* species, especially *C. zhuoniensis* and *C. succinea* [13].

**Figure 4:**
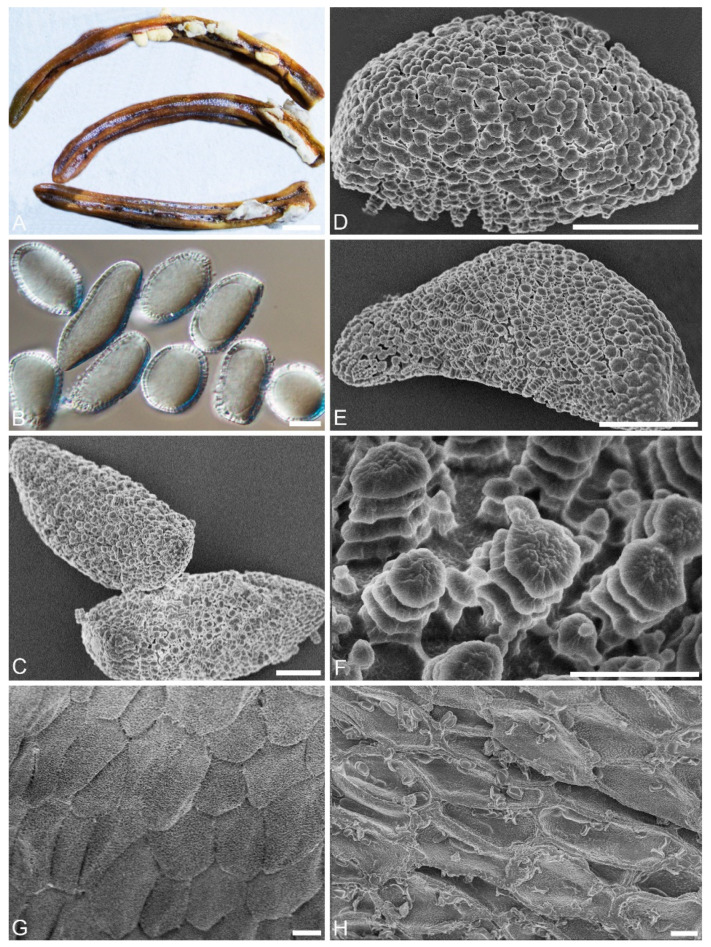
Light and electron micrographs of *Chrysomyxa petalina*. (BJFC-JS145). (**A**) Gross features of infected needles. (**B**) Aeciospores observed by DIC. (**C**) Aeciospores observed by SEM. (**D**,**E**) Single aeciospores with broad, even, annulate warts. (**F**) Ornamentation details of aeciospore. (**G**) Aecial peridium with verrucose inner surface. (**H**) Aecial peridium with oval rugged outer surface. Scale bars: (**A**) = 1000 μm; (**B**–**E**) = 10 μm; (**F**) = 2 μm; (**G**,**H**) = 10 μm.

***Chrysomyxapetalina*** C. J. You and R. Wang, **sp. nov.** (Figure 4)

**MycoBank**: MB 840236

**Specimens examined:** CHINA, Qinghai Province: Guide County, On *Picea crassifolia* Kom., 35°46′29″ N and 101°23′8″ E, 2931 m asl., 3 September 2020, coll. (BJFC-JS145, BJFC-JS146).

**Etymology:** Name refers to the warts with petaloid tops on the aeciospores surface.

Spermogonia, telia and uredinia: unknown.

**Descriptions:** Aecia: On current-year needles of *P. crassifolia*. Aecia discrete, oval or tongue-like (Figure 4A). Aeciospores ellipsoidal, oval, round or long oval, 22–27 × 17–22 μm, with wall 1.9–3.8 μm (Figure 4B); with a reticulated area at one or both ends (Figure 4D); warts cylindrical with thin basal connections, annulate, with petaloid tops (Figure 4C–F). Aecial peridium persistent, cells polygonal, square, polygon or oval, inner surface slightly concave, with distinct and densely crowded warts (Figure 4G). Outer surface shallowly concave, with dense warts (Figure 4H).

**Figure 5:**
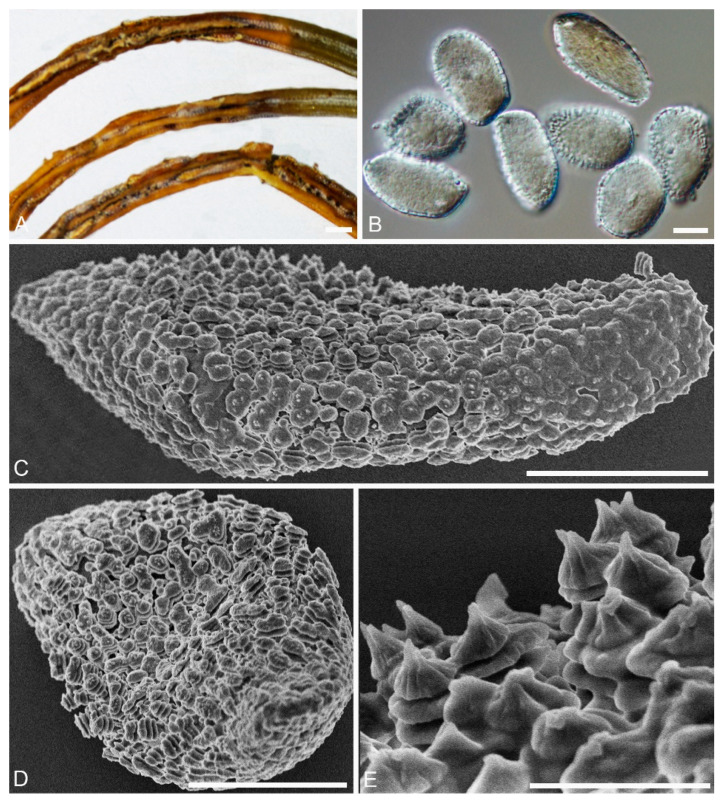
Light and electron micrographs of *Chrysomyxa conituberculata*. (BJFC-MQ02). (**A**) Gross features of infected needles. (**B**) Aeciospores observed by DIC. (**C**,**D**) Single aeciospores, showing densely verrucose warts. (**E**) Warts with three annuli, with a central or split spine. Scale bars: (**A**) = 1000 μm; (**B**–**D**) = 10 μm; (E) =2 μm.

***Chrysomyxa conituberculata*** C. J. You and R. Wang, sp. nov. (Figure 5)

**MycoBank:** MB 840237

**Specimens examined:** CHINA, Qinghai Province: Maixiu forest farm, On *P. crassifolia,* 35°10′29″ N and 101°54′46″ E, 3511 m asl., 3 September 2020, coll. (BJFC-MQ02; BJFC-MQ03).

**Etymology:** Name refer to the conical warts on the surface of aeciospores.

Spermogonia, telia and uredinia: unknown.

**Descriptions:** Aecia: On current-year needles of *P. crassifolia*. Aecia discrete, tongue-like (Figure 5A). Aeciospores oval, round or long oval, 24–31 × 15–20 μm, with wall 1.0–2.4 μm (Figure 5B); with a reticulated area at one or both ends (Figure 5C), annulate warts generally consisting of three or four hemispherical layers, top of the layers in the shape of a conical cap (Figure 5C–E).

**Figure 6:**
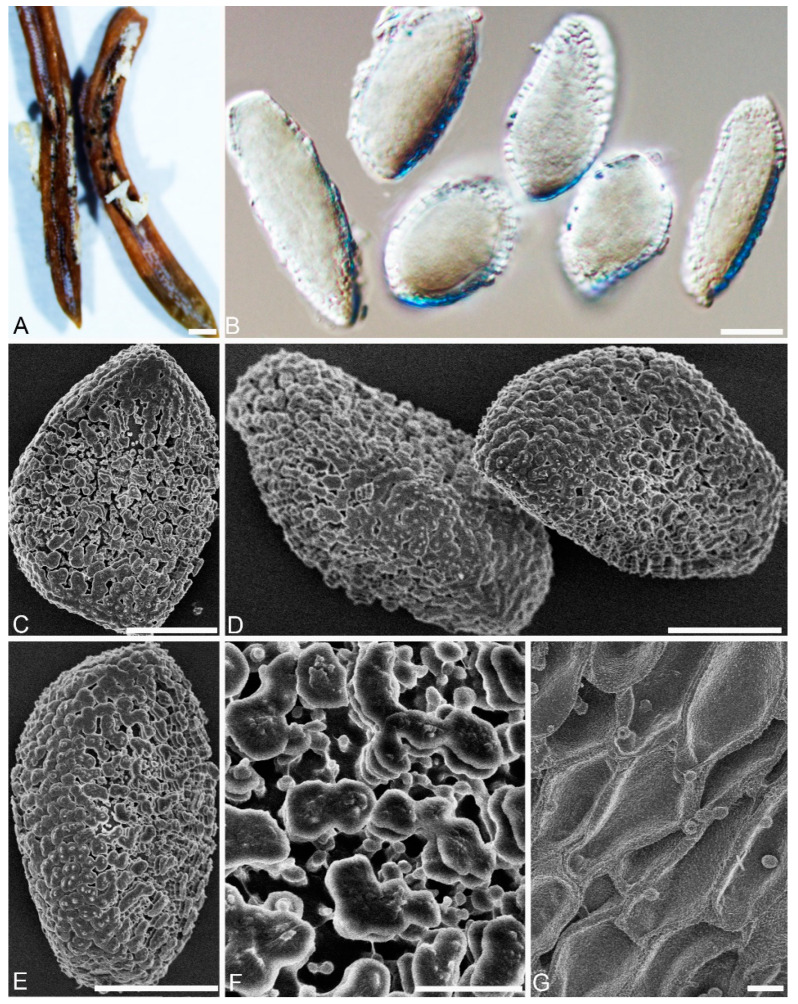
Light and electron micrographs of *Chrysomyxa retiformis*. (BJFC-MQ11). (**A**) Gross features of infected needles. (**B**) Aeciospores observed by DIC. (**D**) Oval aeciospores. (**C**, **E**) Single aeciospore, showing densely and evenly annulate warts. (**F**) Warts with three annuli, with small nodules on the top. (**G**) Aecial peridium with oval rugged outer surface. Scale bars: (**A**) = 500 μm; (**B**–**E**) = 10 μm; (**F**) = 2 μm; (**G**) = 10 μm.

***Chrysomyxa retiformis*** C. J. You and R. Wang, **sp. nov.** (Figure 6)

**MycoBank:** MB 840238

**Specimens examined: CHINA,Qinghai Province:** Maixiu forest farm, On *P. crassifolia*, 35°10′31″ N and 101°54′45″ E, 3523 m asl., 3 September 2020, coll. (BJFC-MQ11, BJFC-MQ08).

**Etymology:** Name refer to the reticulated area on the surface of aeciospores.

Spermogonia, telia and uredinia: unknown.

**Descriptions:** Aecia: On current-year needles of *P. crassifolia*. Aecia discrete, oval or strip-like, 0.17–0.45 mm width, 1.0–4.7 mm long (Figure 6A). Aeciospores oval, round, club-shaped or long oval, 22–47 × 15–24μm, with wall 1.4–2.3 μm (Figure 6B); with a reticulated area over the whole spore surface, part of a broad reticulated area with bumps and broke edges (Figure 6E); warts crowded, annulate, with shallow bumps (Figure 6C–F). Aecial peridium persistent, outer surface oval, shallowly concave, densely covered with warts (Figure 6G).

**Notes:** Only the aecial stage of the three species, *C. petalina*, *C. conituberculata,* and *C. retiformis* are found. *C. petalina* on *Picea* forms a distinct clade sister to *C. rhododendri-capitati.* Morphologically, it differs from *C. conituberculata* in the wall thickness of aeciospores (1.9–3.8 μm vs. 1.0–2.4 μm), and the warts with petaloid tops on spore surface. It differs from *C. retiformis* in the dimension of aeciospores (22–27 × 17–22 µm vs. 22–47 × 15–24μm) and the reticulated area at one or both ends of spore.

**Key to *Chrysomyxa* from China**
1 Telium covered with transparent sheaths21 Telium not covered with transparent sheaths32 Aeciospores have unique echinulate warts
*Chrysomyxa purpurea*
2 Urediniospore densely warted, one side covered by a shallowly warts, longitudinal cap with a ragged edge
*Chrysomyxa forrestii*
3 Only the uredinial stage is found43 Uredinial and aecium stage are found
**6**
4 Urediniospore have verrucose warts
*Chrysomyxa tsukubaense*
4 Urediniospore have not verrucose warts
*5*
5 Urediniospore with a narrow shallow cap at one or both ends, warts crowded, annulate, tower-like, with smooth and rounded tops 
*Chrysomyxa turriformis*
5 Urediniospore without cap 66 Urediniospore not reticulate or smooth 76 Urediniospore reticulate or smooth 87 Urediniospore with densely crowded, narrow, spine-like warts
*Chrysomyxa spinulospora*
7 Urediniospore with annulate, struma-like and fused warts
*Chrysomyxa strumaria*
8 Urediniospore with broad, even, annualte warts 
*Chrysomyxa dumeticola*
8 Urediniospore with variable annulate warts interspersed with smaller warts 
*Chrysomyxa rhododendri-captitati*
9 Aeciospores not smooth or reticulate 109 Aeciospores smooth or reticulate 1310 Aeciospore with nailheaded warts 
*Chrysomyxa dibuensis*
10 Aeciospore without nailheaded warts 1111Aeciospore with single echinae on peltate base; microcyclic
*Chrysomyxa qilianensis*
11 Aeciospore without verrucose warts 1212 Aeciospore with two annuli warts, top of warts smooth and flat 
*Chrysomyxa pyrolae*
12 Aeciospore with three annuli warts, with a central spine on the top of warts
*Chrysomyxa yunnanensis*
13 Aeciospore with cylindrical warts, with three annuli 1413 Aeciospore with cylindrical warts, without three annuli 1514 Aeciospore with a reticulated area at one or both ends, annulate warts generally consisting of three or four hemispherical layers, top of the layers in the shape of a conical cap, Aeciospore 24–31 × 15–20 μm 
*Chrysomyxa conituberculata*
14 Aeciospore with a reticulated area over the whole spore surface, warts crowded, annulate, with shallow bumps, smaller aeciospore 22–47 × 15–24 μm 
*Chrysomyxa retiformis*
15 Aeciospore with annulate warts, with 1–2 annuli, aeciospore 15–43×1439μm
*Chrysomyxa woroninii*
15 Aeciospore with a reticulated area at one or both ends, warts cylindrical with thin basal connections, annulate, with petaloid tops, aeciospore 22–27 × 17–22 μm 
*Chrysomyxa petalina*
16 Aeciospore with a cap1716 Aeciospore without a cap 1917 Aeciospore without a smooth cap 1817 Aeciospore with longitudinal smooth cap, with a broken, fissured edge 
*Chrysomyxa zhuoniensis*
18 Aeciospore with narrow cap, with longitudinal edge 1918 Aeciospore with broad longitudinal cap, with cylindrical warts with smooth or rough tops 
*Chrysomyxa succinea*
Aeciospore with narrow warted groove, outer surface of aecial peridium concave
*Chrysomyxa ledi*
19 Aeciospore with longitudinal smoother area with irregular bumps, inner surface of aecial peridium warted
*Chrysomyxa rhododendri*



## 4. Discussion

### 4.1. Comparison of Species Delimitation Methods

Various studies have compared the species delimitation methods based on single-locus or multi-locus datasets and indicated that different methods often produce incongruent delimitation scenarios [22,46,48,49,50,51,52,53]. To date, there is no similar study conducted on the delimitation of *Chrysomxya* rust species. In our study, three tree-based and one distance-based species delimitation analyses using combined LSU and ITS datasets were conducted, and our results were consistent with many previous empirical studies. Different analyses inferred different group assignments, potentially over- or underestimating the number of putative species. mPTP and bPTP were consistent in recovering fewer OTUs than the number of species originally morphologically considered, and ABGD and GMYC inferred more OTUs than the number of morphological species.

ABGD infers a model-based confidence limit for intraspecific divergence based on prior intraspecific divergences, clustering similar haplotypes together as “species” [28,54]. As in previous studies of empirical data, ABGD is prone to over-lump species, performing slightly worse on more speciose datasets [49,55]. However, ABGD is conservative when using a variety of loci and taxa [27,54,56]. In our study, we considered ABGD as the most conservative method applied to our data, because it recovered the relatively more inferred species and had the highest match ratios. However, in some case, it oversplit some taxonomically accepted species (e.g., *C. zhuoniensis*, *C. rhododendri* from China).

Our bPTP and mPTP analyses produced unreasonable delimitations; some of these clusters do not reflect relationships as understood with better molecular sampling (e.g., *C. arctostaphyli*), and others were separated into numerous lineages despite little-to-no divergence between them (e.g., *C. yunnanensis*). The three tree-based methods employ a coalescent framework based on a single-locus or multi-locus datasets in order to independently identify evolving lineages without gene flow and separate the intraspecific population from interspecific divergence, therefore with each representing a putative species [20,57]. In contrast to ABGD’s underestimation of species diversity, GMYC is known to oversplit species [58,59,60,61]. The results could be affected by higher substitution rates, uneven sampling, variation in population size among species, ongoing gene flow, or unresolved nodes [27,48,49,62,63]. In our study, GMYC recovered more species than distance-based analyses across our data, and bPTP and mPTP inferred fewer species and cannot delimit some genetically distinct lineages. For example, *C. ledi*, *C. ledicola*, *C. neoglandulosi* from Canada, and *C. rhododendri*, *C. ledi* from China, which have been distinguished by morphological characteristics and DNA barcoding [15], were inferred as the same lineage in bPTP and mPTP analyses, but as distinct lineages in ABGD and GMYC.

### 4.2. Species Boundaries

Our analyses (GMYC, bPTP, mPTP, and ABGD), including an expanded morphological and taxonomic sampling, revealed that the rust genus *Chrysomyxa* contains a number of putative cryptic species, and the species diversity in this genus is underestimated.

Two new species, *C. strumaria* and *C. turriformis*, were delimited by all the analyses. As indicated by Dellicour et al. [55], the resulting species delimitation should be correct, if all different types of methods agree with each other. The clade including the nominal species *C. rhododendri-capitati* and *C. dumeticola* showed the existence of cryptic and/or paraphyletic species (Figure 1), and three cryptic species, *C. petalina*, *C. conituberculata,* and *C. retiformis* were recovered by ABGD and GMYC analyses. All the specimens of the three species were collected on *Picea*, and differed in their aeciospore morphology, suggesting that the three lineages identified possibly constitute different species. *C. petalina* formed a sister clade to *C. rhododendri-capitati*, for which only the uredinial stage was found. The aecial–uredinial host connection between the two species should be established by inoculation studies or multiple phylogeny-based and population genetics-based species delimitation analyses.

Some recognized species were inferred by different methods to represent two or more OTUs, each of which appear to represent reciprocally different lineages often separated geographically or by host, for example, *C. qilianensis*, *C. succinea*. *C. zhuoniensis*, *C. dumeticola*, and *C. tsukubaense*. These results suggest that further taxonomic investigation into these lineages, including more thorough molecular sampling, should help to identify ecological processes that drive speciation or even radiation within the genus *Chrysomyxa.*

Geographic variation was apparent in some clusters. For instance, *C. rhododendri* from Canada, Europe, and China should be considered as three cryptic species, although they have similar morphological characters [15]. Our results of all delimitation analyses confirmed that *C. turriforms* was a new species, which differed from other known species in its globose urediniospore with tower-like warts. Interestingly, the teliospores occurring on the two specimens of the new species (BJFC–RA27, BJFC–RA44) were identified as the other known species, the demicyclic species *C. qilianensis.* A previous study has discovered that *C. qilianensis* has a reduced life cycle that lacks the uredinial state [64]. The observed pattern in *C. turriforms*/*C. qilianensis,* occurring on the same leaf in different life state, might be a good example of co-infections caused by many different species on the same host species within a site. It is not uncommon to find that a single host species may be infected by several closely related rust species [65]. For example, the rusts *Uromyces geranii* and *Puccinia leveillei* can be found together on the host plant *Geranium sylvaticum*. Furthermore, a single individual of *Trifolium repens* can be simultaneously infected by two rust species [65,66]. However, the possible mechanism of the observed co-infection pattern, and the possibility of competition between the two rust species are still unclear. Future studies should be carried out on the interactions between two *Chrysomyxa* species and their shared host plant.

## 5. Conclusions

In this study, we utilized a combined LSU and ITS dataset of rust genus *Chrysomyxa* to test the inferences of several common delimitation methods. Although there is some incongruence among tree- and distance-based analyses methods, two new species and three putative cryptic species are identified. Our results suggest that ABGD is the most conservative method in our rust data. In addition, our data demonstrate the unrecognized diversity in need of further taxonomic and systematic investigation. Finally, our work has established a foundation for evolutionary studies of *Chrysomyxa* by providing a framework for species delimitation and efficient identification.

## Figures and Tables

**Table 1:** Substitution saturation tests conducted in DAMBE.

NumOTU ^a^	Iss ^b^	Iss.cSym ^c^	Psym ^d^	Iss.cAsym^e^	Pasym ^f^
4	0.430	0.826	0.000	0.794	0.000
8	0.425	0.797	0.000	0.693	0.000
16	0.491	0.780	0.000	0.588	0.004
32	0.558	0.759	0.000	0.463	0.020

**^a^** numbers of species simulated. **^b^** Index of substitution saturation. ^c^ Index of substitution saturation assuming a symmetrical true tree. ^d^ Probability of significant difference between Iss and Iss.cSym (two-tailed test). ^e^ Index of substitution saturation assuming an asymmetrical true tree. ^f^ Probability of significant difference between Iss and Iss.cAsym (two-tailed test).

**Table 2:** Number of putative species inferred by each delimitation method.

	ABGD ^a^	mPTP ^b^	bPTP ^c^	GMYC ^d^
*N_match_* ^e^	28	20	20	27
*N_delimited_* ^f^	33	21	25	35
Match ratio	0.90	0.80	0.74	0.84

**^a^** Automatic Barcode Gap Discovery. **^b^** Multi-rate Poisson Tree Processes. **^c^** Bayesian implementation of the PTP model. **^d^** Generalized Mixed Yule Coalescent. **^e^** Number of delimited species that exactly match a taxonomically defined morphospecies (not including taxa split as cryptic lineages). **^f^** Total number of lineages delimited by an analysis.

## Data Availability

Data of this study are included in the article or Supplementary Materials.

## Supplementary Materials

The following supporting information can be downloaded at: https://www.mdpi.com/article/10.3390/jof8010083/s1, Table S1: Specimen and sequence data of *Chrysomyxa* used in the molecular analyses in this study.
