# Supplementary material for: Cryptic Species Diversity and Phylogenetic Relationship in the Rust Genus Chrysomyxa from China
Source: J Fungi (Basel). 2022 Jan 15;8(1):83. doi: 10.3390/jof8010083 (PMC8781690; doi:10.3390/jof8010083)
Supplement: Supplementary file 1 [file jof-08-00083-s001.zip › Supplementary Material Table S1.pdf]

Supplementary Material

1 Supplementary Tables

Supplementary Table S1. Specimen and sequence data of *Chrysomyxa* used in the molecular analyses in this study.

Species	Specimen no.	Host	Origin	GenBank or BOLD no.	
				LSU	ITS
C. arctostaphyli	[b]DAOM 183586	*Arctostaphylos uva-ursi*	Kenora district, Ontario, Canada	CHITS053-08	CHITS053-08
	[b]DAOM 229628	*Picea mariana*	Klondike Loop, Yukon, Canada	CHITS040-08	CHITS040-08
C. cassandrae	[b]QFB25005	*Picea mariana*	Abitibi, Quebec, Canada	CHITS052-08	CHITS052-08
	[b]QFB25007	*Chamaedaphne calyculata*	Le'vis, Quebec, Canada	CHITS004-08	CHITS004-08
C. chiogenis	[b]QFB25026	*Gaultheria hispidula*	Charlevoix, Quebec, Canada	CHITS022-08	CHITS022-08
	[b]Only DNA extraction	*Gaultheria hispidula*	Charlevoix, Quebec, Canada	CHITS031-08	CHITS031-08
C. conituberculata	* BJFC-MQ02	*Picea crassifolia* Kom.	Qinghai, China	MZ444055	MZ444048
C. diebuensis	[a]BJFC-R00556	*Picea asperata* Mast.	Gansu,China	MW898417	KX225393
	[a]BJFC-R00507	*Picea asperata* Mast.	Gansu,China	MW063523	KX225394
C. dumeticola	[a]BJFC-R02706	*Rhododendron micranthum* Turcz	Sichuan, China	MK874650	MK770386
	[a]BJFC-R02707	*Rhododendron micranthum* Turcz	Sichuan, China	MK874651	MK770387
C. empetri	[b]QFB25033	*Empetrum nigrum*	Radisson, Quebec, Canada	CHITS032-08	CHITS032-08
	[b]QFB25060	*Empetrum nigrum*	Charlevoix, Quebec, Canada	CHITS033-08	CHITS033-08
C. ledi	[b]DAOM 138900	*Ledum palustre*	Bialowieza Forest, Poland	CHITS056-08	CHITS056-08
	[b]DAOM 162213	*Picea abies*	Pudasjärvi, Jonku, Finland	CHITS059-08	CHITS059-08
	[b]QFB25034	*Picea glauca*	Chisasibi, Quebec, Canada	CHITS028-08	CHITS028-08
	[b]Only DNA extraction	*Ledum groenlandicum*	Waswanipi River, Canada	CHITS060-08	CHITS060-08
	[a]BJFC-R02705	*Picea jezoensis* Carr.	Jilin, China	MK874654	MK770390
C. monesis	[b]DAOM 221985	*Pyrola uniflora*	Graham Island, British	CHITS044-08	CHITS044-08
	[b]DAVFP 10017	*Picea sitchensis*	Columbia, Canada	CHITS107-09	CHITS107-09
C. nagodhii	[b]Only DNA extraction	*Rhododendron groenlandicum*	Manicouagan, Quebec, Canada	CHITS065-08	CHITS065-08
	[b]QFB25054	*Picea mariana*	Charlevoix, Quebec, Canada	CHITS030-08	CHITS030-08
C. neoglandulosi	[b]DAOM 229530	*Ledum glandulosum*	Okanagan, British Columbia, Canada	CHITS042-08	CHITS042-08

C. petalina	* BJFC-JS145	*Picea crassifolia* Kom.	Qinghai, China	MZ444054	MZ444047
C. piperiana	[b]DAFVP 14998	*Ledum macrophyllumc*	Hope, British Columbia, Canada	CHITS113-09	CHITS113-09
C. purpurea	[a]BJFC-R02299	*Picea purpurea* Mast.	Sichuan, China	MW063518	KX225401
	[a]BJFC-R02300	*Picea purpurea* Mast.	Sichuan, China	MW898418	KX225402
C. pyrolae	[b]QFB25056	*Picea sp.*	Bic, Quebec, Canada	CHITS066-08	CHITS066-08
	[b]QFB25055	*Picea glauca*	Lac St-Jean, Quebec, Canada	CHITS013-08	CHITS013-08
	[a]BJFC-R02634	*Picea schrenkiana* Fischet Mey.	Xinjiang, China	MK874655	---
C. qilianensis	[a]BJFC-R02303	*Picea crassifolia* Kom.	Qinghai, China	MZ444062	KX225398
	[a]BJFC-R02100	*Picea crassifolia* Kom.	Qinghai, China	MW898414	MW900425
	*BJFC-RA27T	*Rhododendron przewalskii Maxim.*	Qinghai, China	MW898415	MW900430
	*BJFC-RA44T	*Rhododendron przewalskii Maxim.*	Qinghai, China	MW898416	MW900431
C. retiformis	* BJFC-MQ11	*Picea crassifolia* Kom.	Qinghai, China	MZ444056	MZ444049
C. rhododendri	[b]WM 1183	*Picea abies*	Obere Chlusi, Bernese, Switzerland	CHITS009-08	CHITS009-08
	[b]QFB19829	*Rhododendron ferrugineum*	Simplon, Valais, Switzerland	CHITS036-08	CHITS036-08
	[b]DAFVP 14606	*Ledum lapponicum*	Summit Pass, British, Columbia, Canada	CHITS105-09	CHITS105-09
	[b]DAFVP 14607	*Ledum lapponicum*	Summit Pass, British, Columbia, Canada	CHITS106-09	CHITS106-09
	[a]BJFC-R02712	*Ledum palustre* L	Heilongjiang, China	MK874652	MK770388
	[a]BJFC-R02713	*Ledum palustre* L	Heilongjiang, China	MK874653	MK770389
C. rhododendri-capitati	[a]BJFC-R01054	*Rhododendron lapponicum* (L)Wahlenb.	Qinghai, China	MW063519	MK770382
	[a]BJFC-R02433	*Rhododendron laudandum Cowan.*	Tibet, China	MW898420	MW900429
C. strumaria	* BJFC-RA17	*Rhododendron przewalskii Maxim.*	Qinghai, China	MZ444057	MZ444050
	* BJFC-RA40	*Rhododendron przewalskii Maxim.*	Qinghai, China	MZ444058	MZ444051
C. succinea	[a]BJFC-R02306	*Picea wilsonii* Mast.	Shaanxi, China	MW898419	KX462882
	[a]BJFC-R02307	*Picea wilsonii* Mast.	Shaanxi, China	MK874625	KX462883
	[a]HMAS-6142	*Rhododendron fauriae* Franch.	Japan	---	KX462884
C. tsukubaense	[a]BJFC-R02716	*Rhododendron lepidotum Wall. ex G. Don*	Sichuan, China	MW898412	KY963954
	[a]BJFC-R02720	*Rhododendron lepidotum Wall. ex G. Don*	Sichuan, China	MW898413	MW900428
C. turriformis	* BJFC-RA27U	*Rhododendron przewalskii Maxim.*	Qinghai, China	MZ444059	MZ444052
	* BJFC-RA44U	*Rhododendron przewalskii Maxim.*	Qinghai, China	MZ444060	MZ444053

C. vaccinii	[b]DAOM 45774	*Vaccinium parvifolium*	Graham Island, British, Columbia, Canada	CHITS070-08	CHITS070-08
	[b]DAVFP 18160	*Vaccinium parvifolium*	Victoria Island, British, Columbia, Canada	CHITS115-09	CHITS115-09
C. woroninii	[b]QFB25009	*Ledum groenlandicum*	Charlevoix, Quebec, Canada	CHITS006-08	CHITS006-08
	[b]DAOM 230441	*Picea abies*	Sodankylä, ruosselkä, Finland	CHITS072-08	CHITS072-08
	[a] BJFC-QH02	*Picea crassifolia* Kom.	Qinghai, China	MW898410	MW900423
	[a] BJFC-QH05	*Picea crassifolia* Kom.	Qinghai, China	MW898411	MW900424
C. yunnanensis	[a]BJFC-R02336	*Picea likiangensis* (Franch.) Pritz. var. *linzhiensis* Cheng et L.K.Fu	Tibet, China	MK874644	KY211080
	[a]BJFC-R02337	*Picea likiangensis* (Franch.) Pritz. var. *linzhiensis* Cheng et L.K.Fu	Tibet, China	MK874645	KY211081
C. zhuoniensis	[a]BJFC-R02430	*Rhododendron phaeochrysum* Balf. f. et W. W. Smith	Tibet, China	MW066955	KY963958
	[a]BJFC-R00521	*Picea asperata* Mast.	Gansu,China	MZ444061	KX225396
Melampsora epitea	[ab]US0022745	*Salix sp.*	Abisko, Sweden	AF522170	AY471648

BJFC: Museum of Beijing Forestry University, Beijing, China; BPI: U.S. National Fungal Herbarium; DAOM: Agriculture and Agri-Food Canada, Ottawa, ON, Canada; HMAS: Herbarium Mycologicum Academiae Sinicae, Beijing, China; QFB: Rene Pomerleau Herbarium, Canadian Forest Service, Laurentian Forestry Centre, Quebec, Canada; TSH: Mycological Herbarium of the Graduate School of Life and Environmental Sciences, University of Tsukuba, Japan.

[a] Sequences from the GenBank database.

[b] Sequences from BOLD, and indicates sequences from Feau et al. (2011).

[ab] Sequences used as an outgroup.

*specimens used in this study.

---, Missing information
